# Trends in the Study of Motivation in Schizophrenia: A Bibliometric Analysis of Six Decades of Research (1956–2017)

**DOI:** 10.3389/fpsyg.2018.00063

**Published:** 2018-02-20

**Authors:** Antonia Najas-Garcia, Viviana R. Carmona, Juana Gómez-Benito

**Affiliations:** ^1^Department of Social Psychology and Quantitative Psychology, University of Barcelona, Barcelona, Spain; ^2^Institute of Neurosciences, University of Barcelona, Barcelona, Spain

**Keywords:** intrinsic motivation, co-citation analysis, mapping, negative symptoms, reward, self-efficacy

## Abstract

Motivation in schizophrenia has been a key research aim for several decades. Motivation is a very complex process underlying negative symptoms that has been assessed and identified using very different instruments and terminologies. This study provides a comprehensive overview of the growing literature production and highlights an extensive set of variables to better understand the study of motivation. Electronic databases were searched in order to compile relevant studies of motivation in individuals with schizophrenia. The initial search identified 3,248 potentially interesting records, and of these, 161 articles published between 1956 and 2017 were finally included. Information such as year of publication, journal, country, and number of authors was codified. Variables related to sample characteristics, methodological aspects, and motivational terms were also extracted. The results revealed a significant growth trend in literature production, especially since the 2000s, with reward as the main term studied. In addition, questionnaires were identified as the preferred instrument to assess motivation in patients with schizophrenia. Other aspects such as country of publication, authors, journals of publication, and co-citation network analysis were also examined. The discussion offers recommendations for future research.

## Introduction

Interest in motivation in schizophrenia has increased considerably in recent years (Gard et al., 2009; Kremen et al., 2016). Most studies have defined motivation as a core negative symptom in schizophrenia that is related to poor functional outcome (Fervaha et al., 2015; Foussias et al., 2015). One of the most recent conceptualizations delineates two possible sub-domains of negative symptoms in schizophrenia: diminished expression and motivational deficits (Blanchard and Cohen, 2006; Foussias and Remington, 2010; Messinger et al., 2011). Diminished expression involves blunted affect and poverty of speech, while motivational deficits refer to a decrease in goal-directed behavior and the associated internal processes, such as curiosity, interest, and drive, which prompt the individual to plan, initiate, and pursue activities (Andreasen, 1982; Nakagami et al., 2008). The latter domain typically involves avolition/apathy and anhedonia/asociality (Kelley et al., 1999; Kimhy et al., 2006), and it is usually referred to as apathy (Hartmann et al., 2015) or avolition (Foussias and Remington, 2010).

For many years, it was thought that patients with schizophrenia failed to pursue goal-directed activities because they did not find such activities enjoyable, and thus anhedonia was considered to be a key negative symptom of schizophrenia (Strauss, 2013; Strauss et al., 2014). However, recent findings question the presence of anhedonia in schizophrenia because it has been found that individuals with this illness report intact consummatory pleasure, comparable to that of healthy controls (Horan et al., 2006; Foussias and Remington, 2010; Strauss and Gold, 2012). Despite these preserved hedonic responses, however, it is clear that individuals with schizophrenia less frequently engage in motivated behaviors aimed at obtaining rewards and pleasurable outcomes (Myin-Germeys et al., 2000). In this context, a prominent line of research has indicated that motivational deficits can reflect disordered processing of rewards and value-based decision-making (Juckel et al., 2006; Strauss et al., 2013, 2014). It is also possible that the motivational deficits of patients with negative symptoms reflect low expectations about performing activities successfully (Bentall et al., 2010). Indeed, from the perspective of the cognitive model (Rector, 2004; Rector et al., 2005) it has been suggested that dysfunctional attitudes such as defeatist performance beliefs contribute to the development of negative symptoms and, consequently, to a lack of goal-directed behavior. Whatever the case, the study of motivation in schizophrenia is clearly important because it has been shown to have a significant influence on areas such as psychosocial functioning (Nakagami et al., 2010), quality of life (Buck and Lysaker, 2013), work outcome (Reddy et al., 2016), and cognitive function and treatment adherence (Campellone et al., 2016; Fiszdon et al., 2016).

Despite the long history of research on motivation in schizophrenia and the volume of literature available, there have been few reviews and bibliometric studies of this topic. The reviews which have been published have focused on the main measures used to explore apathy (Bortolon et al., 2017), the association between reward processing and motivational impairment (Strauss et al., 2014), or developments in the concept of apathy (Del-Monte, 2013). As for bibliometric studies, these have examined trends in the literature on antipsychotics (López-Muñoz et al., 2015), production of neurocognition literature in schizophrenia (Guilera et al., 2012), production of schizophrenia literature compared with the total medical literature (Theander and Wetterberg, 2010), and patient-centered medicine (Calton et al., 2009).

Given the relevance of motivational deficits to recovery and their association with negative functional outcomes, such as the inability to secure employment or to conduct normal activities of daily living (Cardenas et al., 2013), we believe it is important to examine, through an exhaustive search and bibliometric methods, the different terms used in studies related to motivation in schizophrenia. Bibliometric studies are useful tools for systematically assessing and analyzing research publications, and hence for evaluating the social and scientific relevance of a given discipline or field (Koskinen et al., 2008). Typically, a bibliometric study analyzes the evolution of scientific production though different indicators, including: quantity indicators, which measure the productivity of a particular researcher or research group; structural indicators, which measure connections between publications, authors, or research fields; and performance indicators, focused on the quality of a journal, researcher, or research group (Durieux and Gevenois, 2010). Accordingly, the aim of the present study was to identify current trends in, and the scope of, research on motivation in schizophrenia and to explore both how these trends have evolved over time and the relationship between them. To this end, we present descriptive information regarding the studies identified, including sample characteristics, terminology, and the instruments used to examine motivation. The value of a study of this kind is that it can illustrate the current research efforts being made in the field and help to achieve a better understanding of the main processes related to motivation. The qualitative information provided and the comprehensive picture obtained of the field of study make it a robust and effective complement to expert appraisals in this field.

## Methodology

Studies were compiled from the Web of Science platform by searching in the *Medline* and *Web of Science TM Core Collection* databases. These databases were chosen due to their broad and encompassing content regarding severe mental illness, including the characteristics, functioning, and recovery of patients. The study was conducted in accordance with the PRISMA Statement on preferred reporting items for systematic reviews and meta-analyses (Moher et al., 2010), as described below.

### Search strategy

To collect potentially relevant articles for the study, a systematic search was conducted using Boolean operators between the following key words (1) Title: psychosis OR schizophrenia OR schizoaffective disorder; AND (2) Topic: motivation^*^ OR apathy OR self-efficacy OR reward OR reinforcement OR avolition. The searches were conducted without language restriction from 1950 until 29 November 2017 in Medline, and from 1900 until 29 November in Web of Science. The same search strategy was applied to both databases.

### Exclusion and inclusion criteria

Duplicate items between the two databases were excluded, as were reviews, meta-analyses, books and book chapters, qualitative studies, narrative studies, editorial material, abstracts, agreements, theoretical articles, proceedings of meetings, historical papers, studies not related to motivation, studies with samples of patients at high risk of schizophrenia, studies of subjects under 18 years old, and studies involving first episode of psychosis, animals, healthy subjects, or families of schizophrenia patients.

Given the objectives of this study and the great diversity of documents addressing schizophrenia and motivation, only original articles published in journals were considered. The overall sample consisted of patients diagnosed with schizophrenia, in some cases together with other psychotic disorders. Diagnoses were made using standard manuals such as any edition of the Diagnostic and Statistical Manual of Mental Disorders (American Psychiatric Association, 1994), the Schedule for Affective Disorders and Schizophrenia (Endicott and Spitzer, 1978), or the International Classification of Diseases (World Health Organization, 1977).

### Data collection

Studies were screened by title and abstract prior to reviewing the full text. The data extracted were tabulated in Excel 2007. The coded variables were as follows:

Terms used to refer to motivation: (1) intrinsic motivation [IM]; (2) self-efficacy and defeatist beliefs (SE); (3) apathy (AP); (4) rewards or reinforcements (RR); (5) avolition (AV); and (6) other motivation terms (O), where studies examined different and independent terms that could not be included in any prior conceptualization. Reward variables were also coded, given their theoretically close involvement in motivation (Fervaha et al., 2013).

Studies were also coded if they used: (1) brain techniques, (2) behavioral tasks, (3) questionnaires/self-reports, or (4) other instruments to measure motivation. Additionally, studies were coded on the basis of variables related to the sample characteristics, such as number of participants, sex, mean age, ethnicity, diagnosis, illness duration, and educational level. Variables such as the year of publication, the number of authors, the countries involved in the study, and the journal in which the article was published were also coded.

All variables were coded by two members of the research team and discrepancies were resolved through discussion with a third expert. Inter-rater reliability was high across variables [Cohen's kappa (Cohen, 1968) = 0.801 for categorical variables; intra-class correlation (Shrout and Fleiss, 1979) = 0.992 for continuous variables].

### Data analysis

Given that our main focus was quantity bibliometric indicators and structural bibliometric indicators (Durieux and Gevenois, 2010), we used descriptive statistics to examine the main variables. The analyses performed focused on frequencies and percentages of articles regarding: terms used to refer to motivation, methodology used to study motivation, sample characteristics, and countries. The countries of origin of all authors were taken into account, so several studies were classified as pertaining to more than one country. Additionally, two laws were applied: Price's law (Price, 1963) and Lotka's law (Lotka, 1926). Price's law is the most widely used indicator for analyzing the productivity of a specific discipline or a country, and it reflects a fundamental aspect of scientific production, namely its exponential growth (López-Muñoz et al., 2006). Lotka's law assumes that there are many authors who publish only one study, while a small group of prolific authors contribute with a large number of publications (Lotka, 1926). In order to apply Lotka's law, the complete count of first authors and co-authors was included in the analysis. Data were analyzed using the Statistical Package for the Social Science (SPSS for Windows 22.0).

Since the prospective links between motivation and schizophrenia integrate findings from disparate disciplines, we analyzed co-citation patterns to identify cross-disciplinary collaboration in the studies examined (Börner et al., 2003, 2007; Van Eck and Waltman, 2014; Barlow et al., 2017). Co-citation analysis is the most commonly used bibliometric method (Ding et al., 2014) and it measures the frequency with which two documents are cited together by other documents. This enabled us to assess whether insights from different fields are being mutually acknowledged or are instead confined to different disciplinary domains. We also evaluated the relationship between cited references based on the co-occurrence of references within articles. Co-citations represent a link between two documents, indicated by references and by the first authors of articles. Thus, if two authors or references were cited in the same paper, it meant that they were closely related. To map co-citation patterns, we extracted citation data from Web of Science and analyzed it using VOSviewer 1.6.5., a software package for constructing and visualizing bibliometric maps (Van Eck and Waltman, 2009, 2010). VOSviewer is a tool that takes a distance-based approach to visualizing bibliometric networks, such that the distance between two nodes offers an approximate indication of their relatedness, a feature which makes the software suitable for visualizing larger networks (Van Eck and Waltman, 2014). VOSviewer applies by default a measure of association to normalize the data, known as the VOS mapping technique, where VOS stands for “visualization of similarities” (Van Eck and Waltman, 2009; Van Eck et al., 2010). The VOS technique also uses an algorithm to assign nodes in a network of clusters, where a cluster is a set of closely related nodes (Van Eck and Waltman, 2009, 2014). The number of clusters is determined by a resolution parameter in the algorithm, and the higher the value of this parameter, the larger the number of clusters (Waltman et al., 2010). Typically, the researcher has to set a threshold regarding the frequency of the references contained in the analysis, leaving out cited documents that do not have a significant impact (Milojević, 2014; Flis and van Eck, 2017). Given that the literature does not offer guidance on how to select a particular threshold level, in this study a citation frequency threshold was chosen by investigating citer–cited networks with different thresholds in order to find an appropriate level that excluded only less related documents (Schildt et al., 2006).

## Results

### Search results

**Figure 1:**
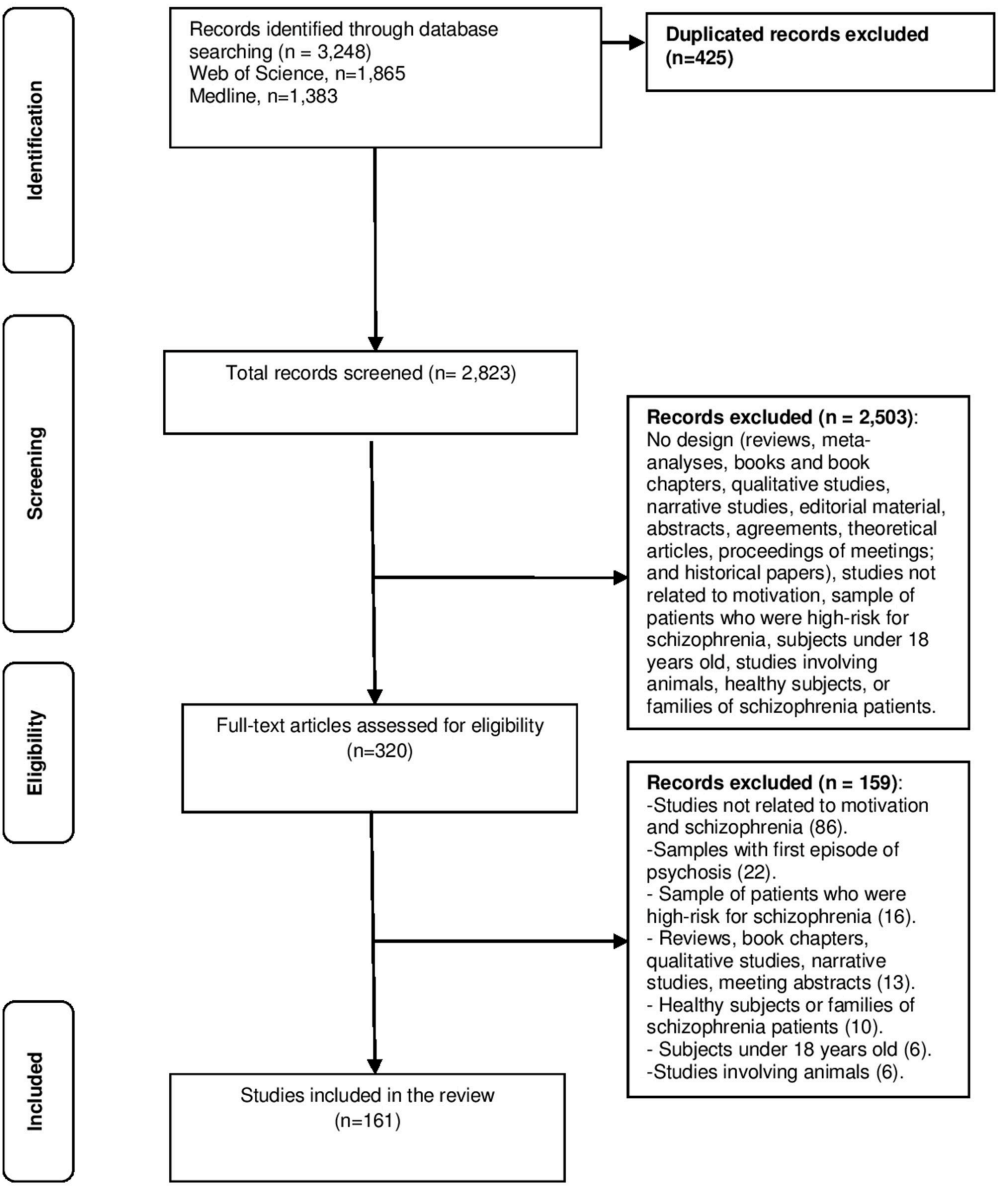
Search strategy used, following a PRISMA flow diagram.

Figure 1 shows the four-phase flow diagram based on the PRISMA Statement (Liberati et al., 2009). The search retrieved 3,248 studies, of which 2,823were considered for inclusion after removal of 425 duplicates. Initial screening excluded 2,503 of these articles, and in the remaining 320 cases the full article was screened. Of these, 159 records were excluded for the following reasons: studies not related to motivation (86); sample comprised people who were high-risk for schizophrenia, healthy subjects, or families of schizophrenia patients (26); studies including samples with first episode of psychosis or subjects under 18 years old (28); reviews, book chapters, qualitative studies, narrative studies, meeting abstracts, and studies in animals (19). The remaining 161 studies were included in the present study.

### Characteristics of studies included

#### Sample characteristics

The studies examined comprised a total sample of 14,239 participants: 2,997 control subjects, 10,192 people diagnosed with schizophrenia, 582 people with schizoaffective disorder, and 196 with other mental disorders. The total sample was predominantly male (61.89%), with a mean age of 38.49 years. More than half of the participants were white/Caucasian (62.53%), with a mean of 13.64 years of education. See Table 1 for details.

**Table 1:** Sample characteristics by groups.

	**Total (*n* = 161)**	**Control (*n* = 61)**	**Schizophrenic (*n* = 161)**	**Schizoaffective (*n* = 62)**	**Other disorders (*n* = 16)**
Number of participants	14,239	2,997	10,192	582	196
Mean age	38.49	34.93	36.77	44.12	38.16
% Ethnicity	62.53	59.53	56.98	49.67	83.93
% Male	61.89	61.63	66.19	68.38	51.36
Mean education (years)	13.64	14.11	12.89	13.37	14.17
Mean illness duration (years)	13.20		13.20		

*n, number of studies; ethnicity, % Caucasian*.

### Terminology

Twenty-seven studies considered more than one term for motivation. The most frequently used term across all studies was RR (in 84 studies; 52.17% of the sample), followed by IM (28; 17.39%), AP (27; 16.77%), SE (17; 10.55%), and AV (15; 9.32%). Nine studies (5.59%) examined motivation under other conceptualizations that could not be categorized.

### Techniques of assessment

Most of the studies (99; 61.49%) used questionnaires to assess motivation. These were followed by behavioral tasks (96; 59.63%) and brain techniques (46; 28.57%).

Sixty-two of the studies combined two or more techniques to examine motivation in patients with schizophrenia, so more than one methodology may be recorded for each study. Table 2 shows a classification of the measurement instruments used according to the different motivation terms. Studies of RR were carried out predominantly with behavioral tasks and brain techniques, while studies of IM and AP mainly used questionnaires.

**Table 2:** Measurement instruments used to assess the different motivation terms.

**Term**	**Brain techniques**	**Behavioral tasks**	**Questionnaires**
RR	36	74	28
IM	2	6	26
AP	7	13	27
SE	0	2	17
AV	1	5	11
O	1	3	8

*AP, Apathy; AV, Avolition; IM, Intrinsic Motivation; RR, Rewards or Reinforcements; SE, Self-Efficacy and Defeatist Believes; O, other terms of motivation*.

### Scientific production on motivation in schizophrenia

#### Productivity by country

**Figure 2:**
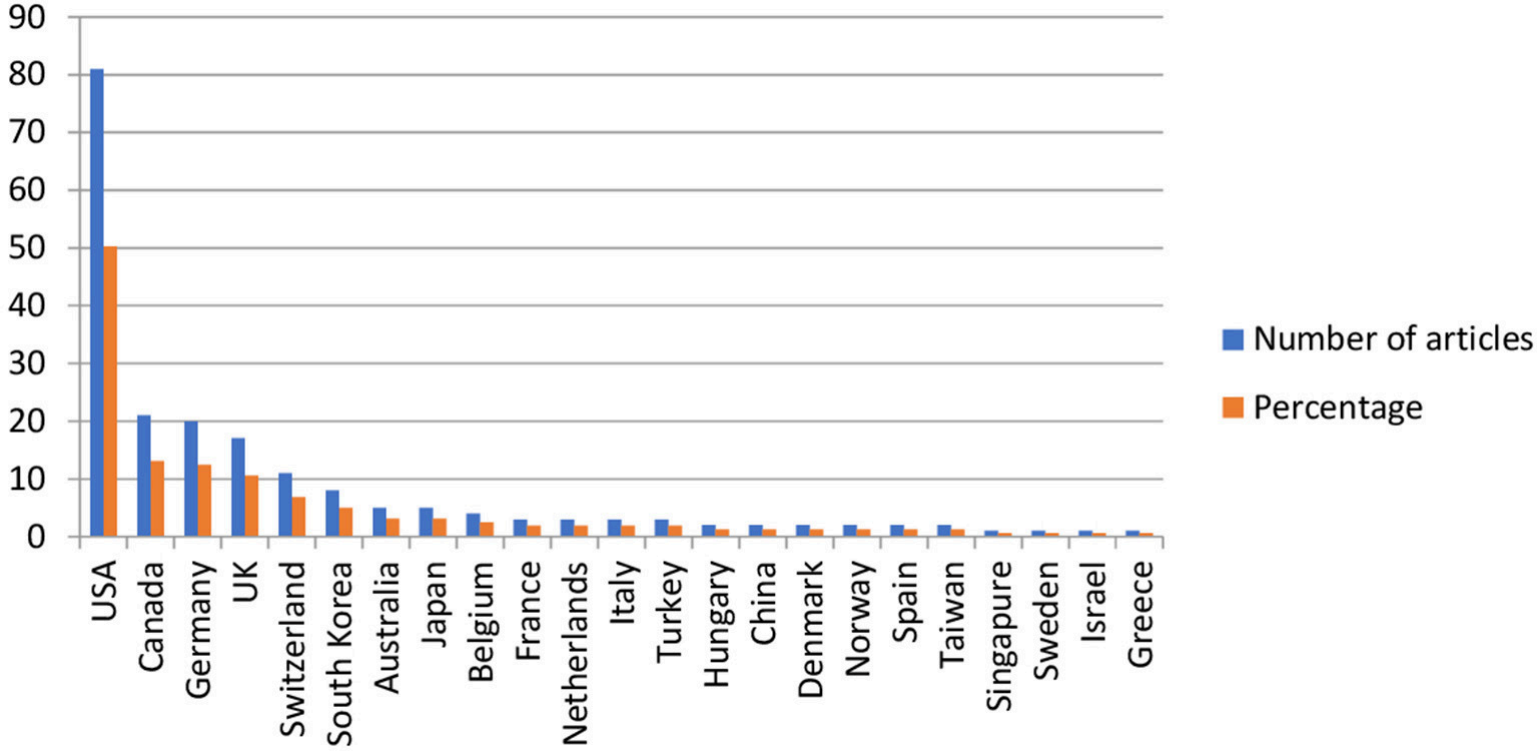
Contribution by countries.

Twenty-three countries were represented in the literature. Half of the studies (50.31%) were from the USA (81), 13.66% from Canada (22), 12.42% from Germany (20), 10.55% from the UK (17), 6.83% from Switzerland (11), 4.97% from South Korea (8), and 3.10% from each of Australia (5) and Japan (5). All remaining countries contributed less than 2% of the studies (fewer than 5 articles) (see Figure 2).

Thirty-five studies (15.84%) were international collaborations. The USA was represented in 20 collaborative studies, the UK in 12, and Canada and Germany in six.

#### Productivity of journals

The articles included were published in 57 different journals, although many were published in a small number of specialized journals. The four journals that published the most studies of motivation in schizophrenia were Schizophrenia Research (46 published studies), Psychiatry Research (18), Schizophrenia Bulletin (12), and the Journal of Abnormal Psychology (8). Together, they accounted for 52.17% of the articles identified.

#### Productivity of authors

A total of 622 authors contributed to the output in this study. Some contributed to more than one study; the most productive author (G. Remington) was involved in the publication of 13 articles, while the five most productive authors contributed to more than 47.82% of the total publications. The mean number of authors per article was 5.50 (SD = 2.23). The data showed that only 1.86% of the articles had a single author (3), 6.21% had two (10), 11.80% had three (19), 16.77% had four (27), a further 16.77% had five (27), and 46.59% had six or more authors. With regard to the productivity of authors, 75.08% of them contributed one article. As noted earlier, Lotka's law describes the productivity distribution among scientists and states that a small group of researchers is responsible for most of the literature, whereas the majority contribute a very small number of publications. In the articles inspected, 467 authors contributed only one article, 106 contributed two, 23 contributed three, and nine contributed four. Lotka's law was evaluated considering all authors of the publications (first authors and collaborators). To determine whether the data fitted Lotka's law, the *n* value was calculated using the least squares method (*n* = −2.60, obtaining a *C* value of 0.7866). The critical value obtained by the non-parametric Kolmogorov-Smirnov goodness-of-fit test was 0.0019. As the maximum difference between the observed and the estimated accumulated frequencies was 0, which is below the critical value (0.0019), we can conclude that the data fitted Lotka's law.

### Network analyses of journals, citations, authors, and terms

**Figure 3:**
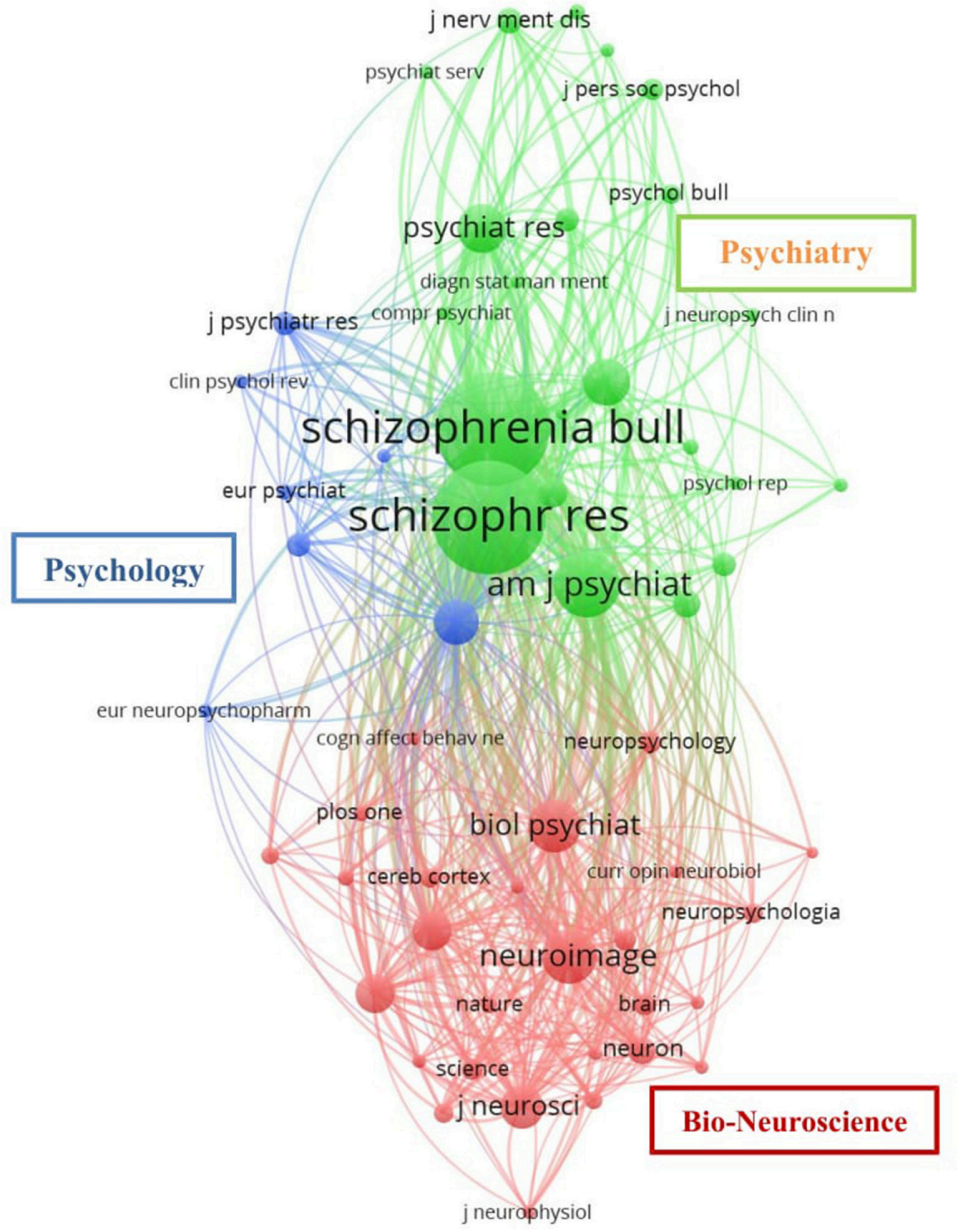
Network resulting from the co-citation analysis by journal. Co-citation patterns among 56 journals cited at least 20 times from 1956 to 2017. Each node represents a journal, node size reflects the relative contribution of each journal. Map with full counting method, association strength normalization and default clustering resolution (1.00).

Figure 3 shows the results from our co-citation analysis by journal. The network map shows co-citation patterns of 56 of the 1,000 journals that were cited at least 20 times within the studies we identified. Node size corresponds to the number of citations, lines correspond to the existence of a citation in either direction, and distance between nodes corresponds to the tendency for studies to be cited together by other studies. The color of nodes indicates the cluster to which a journal was assigned by the clustering algorithm. After inspecting the nodes of which each cluster was comprised, we manually assigned a descriptive label based on the thematic content. The first cluster, involving journals with publications on topics such as neuroimaging, neuroscience, and cognition, was labeled the “Bio-neuroscience” cluster (red); a second cluster including journals publishing on psychotherapy, psychiatric illness, and normal human behavior was labeled the “Psychology” cluster (blue); finally, a third cluster that predominantly included psychiatry research was labeled the “Psychiatry” cluster (green).There was a strong tendency for co-citation of studies in the psychiatry cluster, and to a lesser extent in the “Bio-neuroscience” cluster.

**Figure 4:**
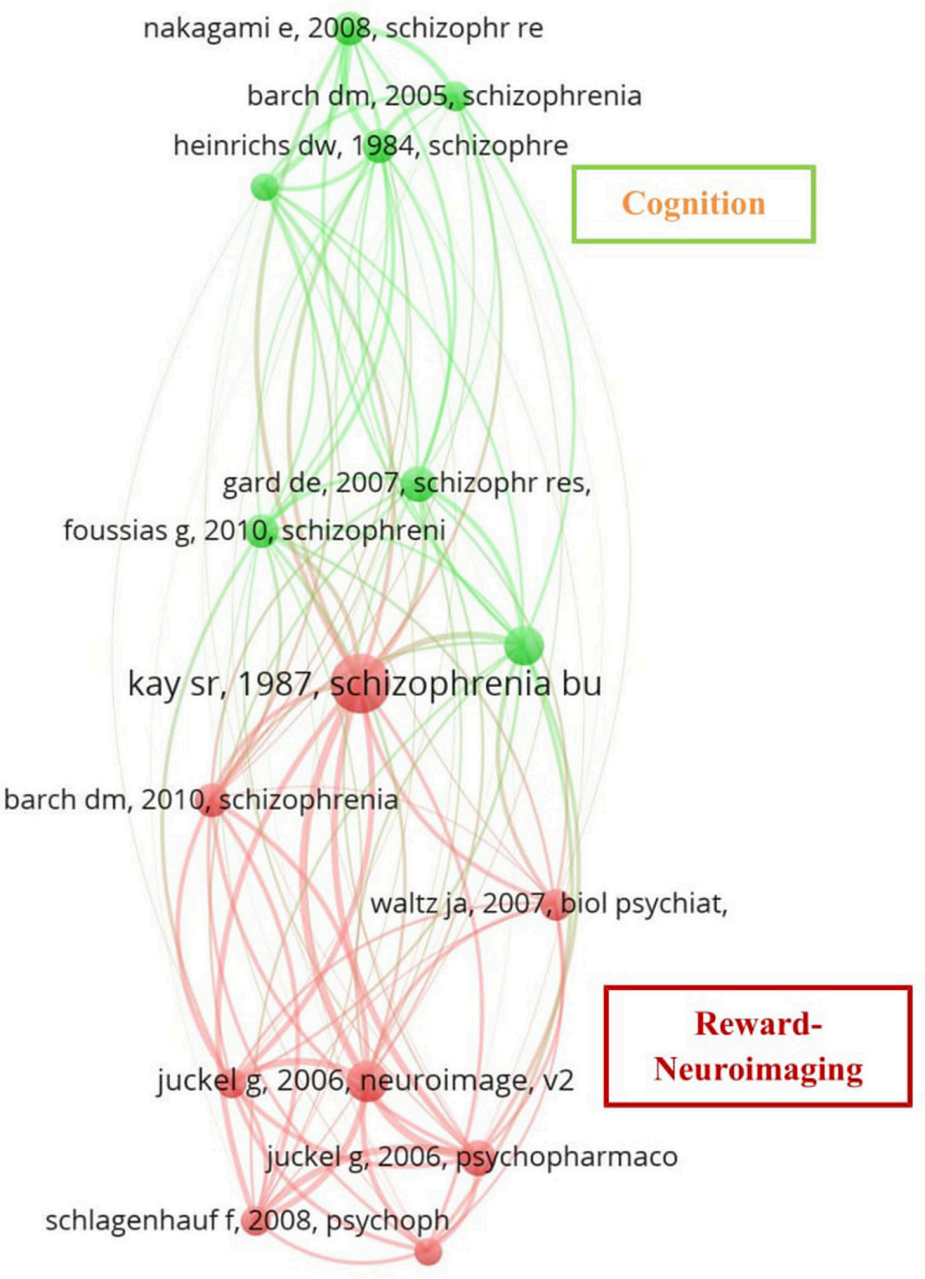
Network resulting from the co-citation analysis by cited reference. Co-citation patterns among 15 references co-cited at least 20 times published between 1987 and 2010. Each node represents a paper, node label is the last name of the first author and the journal where the work was published, edges represent citation relations, node color represents the cluster to which a reference belongs. VOSviewer map with full counting method, association strength normalization, and default clustering resolution (1.00).

Co-citation analysis by cited references revealed that the 161 articles selected for analysis had 3,972 cited references. Our search for a threshold level where the citing documents remained constant included 15 references cited at least 20 times. The network resulting from these analyses can be seen in Figure 4, which shows the clustered network of the 15 most frequently cited publications in the collection examined, where a cluster being a set of strongly connected publications in terms of co-citation relations. The clusters formed suggest a clear split of topics that mainly correspond to studies on cognition and schizophrenia symptoms, labeled “Cognition” in the figure (green), and studies on reward/neuroimaging, labeled “Reward/Neuroimaging” (red). An overview of the 15 references included in the network, with the number of co-citations and strength of the link, is shown in Table 3.

**Table 3:** Co-cited references included in the network analysis.

**Cited references**	**Citations**	**Total link strength**
Kay SR (1987), *Schizophr. Bull*, v13, p261, doi 10.1093/schbul/13.2.261	60	191
Juckel G (2006), *Neuroimage*, v29, p409, doi 10.1016/j.neuroimage.2005.07.051	37	147
Juckel G (2006), *Psychopharmacology*, v187, p222, doi 10.1007/s00213-006-0405-4	29	133
Gold JM (2008), *Schizophr. Bull*, v34, p835, doi 10.1093/schbul/sbn068	34	132
Barch DM (2010), *Schizophr. Bull*, v36, p919, doi 10.1093/schbul/sbq068	27	119
Simon JJ (2010), *Schizophr Res*, v118, p154, doi 10.1016/j.schres.2009.11.007	23	118
Foussias G (2010), *Schizophr Bull*, v36, p359, doi 10.1093/schbul/sbn094	28	109
Schlagenhauf F (2008), *Psychopharmacology*, v196, p673, doi 10.1007/s00213-007-1016-4	23	109
Gard DE (2007), *Schizophr Res*, v93, p253, doi 10.1016/j.schres.2007.03.008	29	107
Heinrichs DW (1984), *Schizophrenia Bull*, v10, p388, doi 10.1093/schbul/10.3.388	26	101
Nakagami E (2008), *Schizophr Res*, v105, p95, doi 10.1016/j.schres.2008.06.015	27	97
Waltz JA (2009), *Neuropsychopharmacol*, v34, p1567, doi 10.1038/npp.2008.214	20	95
Gard DE (2009), *Schizophr Res*, v115, p74, doi 10.1016/j.schres.2009.08.015	21	89
Waltz JA (2007), *Biol Psychiatry*, v62, p756, doi 10.1016/j.biopsych.2006.09.042	25	88
Barch DM (2005), *Schizophr Bull*, v31, p875, doi 10.1093/schbul/sbi040	23	77

**Figure 5:**
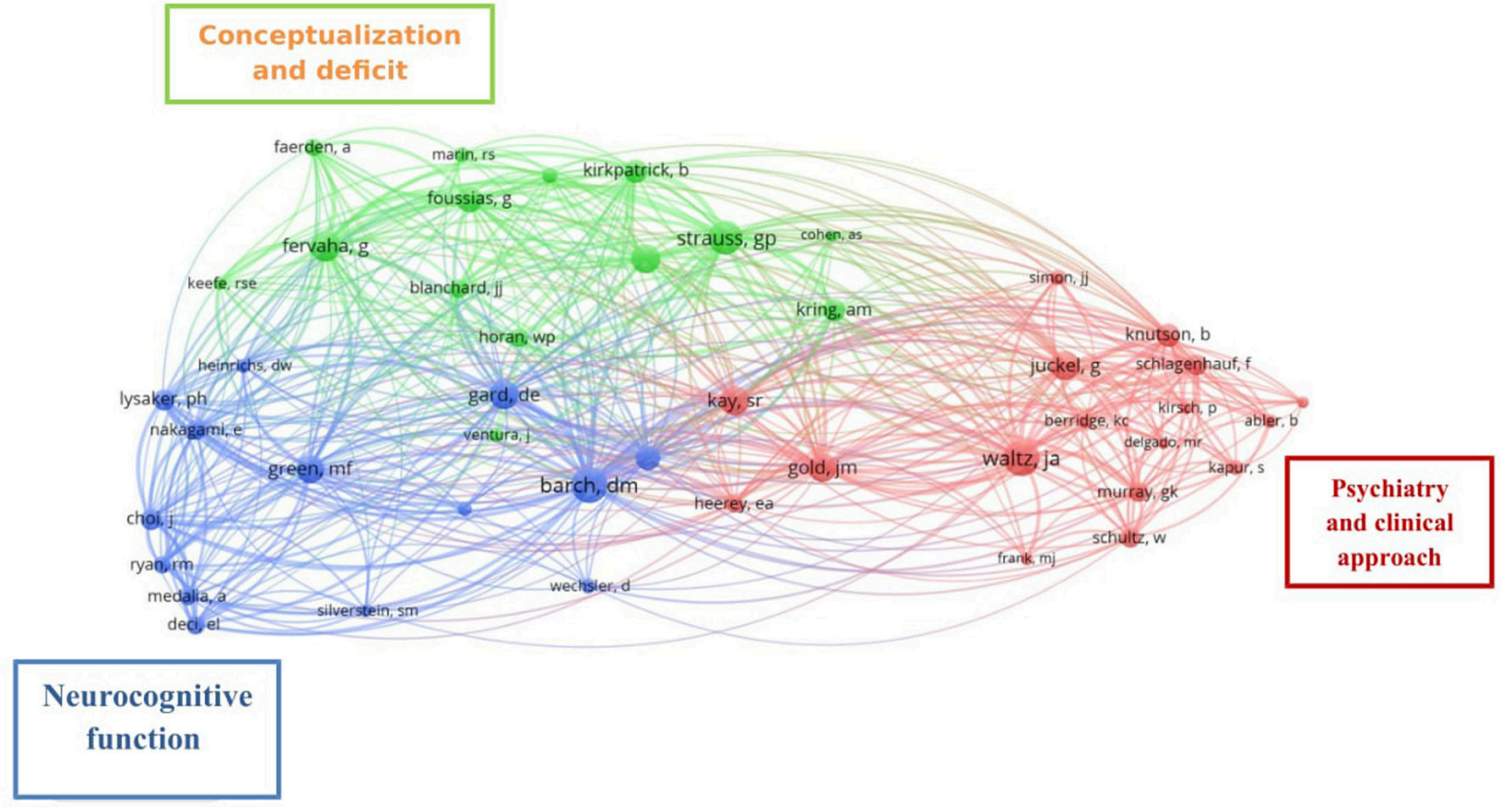
Network resulting from the co-citation analysis by the author. Co-citation patterns of 45 authors cited at least 20 times in the literature between 1956 and 2017. Each node represents an author, node label is the last name of the first author, and edges represent citation relations. Map with full counting method, association strength normalization, and default clustering resolution (1.00).

The co-citation analysis by author included 2,564 cited authors, 45 of whom were cited at least 20 times. Figure 5 depicts the network resulting from this analysis: each color represents a community of authors within the same subject of interest, and authors within a cluster represent a set of strongly connected authors in terms of co-citation relations. One of the notable communities (show in red) includes the authors JA Waltz, G Juckel, JM Gold, and SR Kay, who have been addressing motivational studies from a psychiatric and clinical perspective and examining negative symptoms though scales and computerized systems, which we label “Psychiatry and clinical approach.” Another group (shown in blue), comprising DM Barch, DE Gard, and MF Green, has addressed motivation from the neurocognitive and social cognition perspectives, examining its implications for daily living and functionality, which we label “Neurocognitive function.” Finally, the group (shown in green) involving GP Strauss, G Fervaha, G Foussias, and B Kirkpatrick has studied motivation with respect to the conceptualization and description of deficit syndrome, reward processing, and therapeutic approaches for addressing this, which we label “Conceptualization and deficit syndrome.” Table 4 shows the names of the 45 authors included in the network, with the corresponding number of co-citations and strength of the link.

**Table 4:** Co-cited authors included in the network analysis.

**Author**	**Citations**	**Total link strength**
Barch, DM	93	1,687
Strauss, GP	83	1,535
Waltz, JA	91	1,492
Fervaha, G	73	1,417
Gard, DE	70	1,368
Gold, JM	70	1,294
Juckel, G	70	1,080
Foussias, G	57	1,042
Andreasen, NC	68	977
Green, MF	64	967
Kay, SR	68	956
Kirkpatrick, B	51	901
Kring, AM	48	882
Choi, J	45	847
First, MB	54	840
Knutson, B	52	833
Nakagami, E	40	822
Schlagenhauf, F	44	811
Lysaker, PH	45	732
Murray, GK	41	692
Blanchard, JJ	38	686
Ryan, RM	34	658
Heerey, EA	35	651
Schultz, W	39	646
Medalia, A	35	638
Deci, EL	36	612
Horan, WP	37	581
Faerden, A	33	557
Heinrichs, DW	26	532
Addington, D	29	508
Keefe, RSE	23	476
Simon, JJ	27	466
Berridge, KC	26	465
Delgado, MR	20	434
Kapur, S	28	432
Ventura, J	24	409
Kirsch, P	23	393
Abler, B	24	392
Silverstein, SM	23	392
Cohen, AS	20	382
Wechsler, D	27	372
Marin, RS	29	366
American psychiatric association	25	351
Frank, MJ	20	338
Heinz, A	20	276

**Figure 6:**
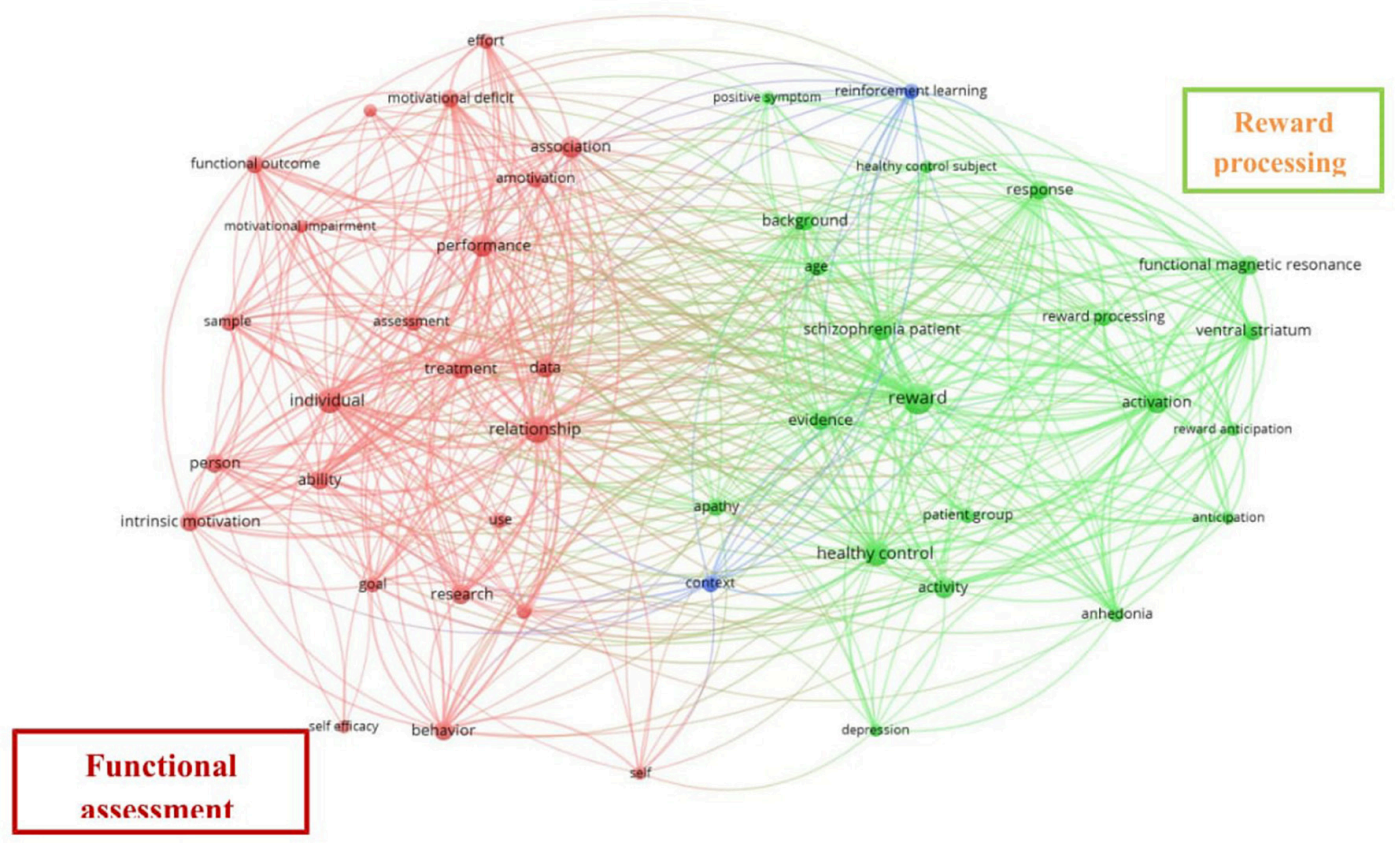
Co-occurrence of terms. Map with binary counting method, association strength normalization, and default clustering resolution (1.00).

With regard to the concepts and definitions of motivation used in research on schizophrenia, we also analyzed networks of co-occurrences of terms. To this end, we scanned the 161 publications in the dataset (Web of Science output files) using VOSviewer (Van Eck and Waltman, 2009) to extract noun phrases from their titles and abstracts. Figure 6 provides a visual representation of the relationship between frequently co-occurring terms used in the study of motivation in schizophrenia; the list of terms was derived using a natural language processing algorithm to exclude verbs, adverbs, adjectives, conjunctions, etc. We used a binary counting method, that is, the co-occurrence frequency of two terms. Since VOSviewer asks for the minimum number of occurrences a noun phrase must achieve in order to be included in the co-occurrence network. Here, we chose a default value of 10 occurrences, meaning that terms would be included if they appeared at least 10 times in the title and abstract fields. The analysis identified 3,377 terms, of which 76 met the threshold of at least 10 occurrences. Of these 76 terms we selected by default the most relevant 60%, such that the final network comprised 46 terms. In the depicted network the size of the nodes denotes the frequency of a term, its proximity with other terms indicates the degree of relatedness with other terms, and the node color represents the cluster to which a term belongs. Results from the analysis divide the terms into two main clusters and a third marginal cluster. The two main clusters refer to (1) Reward processing terms and other associated terms that constitute different methods involved in their study (shown in green), which we label “Reward processing,” and (2) different terms associated with ability, assessment, performance, and treatment, which we label “Functional assessment” (shown in red). A full list of the 46 most relevant terms extracted from papers in the dataset with at least 10 occurrences is given in Table 5.

**Table 5:** Co-occurrence of terms in the network analysis.

**Term**	**Occurrences**	**Relevance score**
Anticipation	11	29.959
Reward anticipation	10	28.492
Ventral striatum	20	25.309
Functional magnetic resonance imaging	21	24.593
Functional outcome	19	24.208
Intrinsic motivation	20	19.635
Activation	29	19.269
Sample	17	18.479
Person	22	14.793
Reward processing	16	13.995
Anhedonia	14	13.649
Motivational impairment	10	10.439
Goal	13	10.423
Individual	35	10.133
Self efficacy	10	2.063
Baseline	10	0.972
Behavior	20	0.8898
Assessment	17	0.8871
Response	21	0.8819
Depression	11	0.8191
Schizoaffective disorder	15	0.7878
Motivational deficit	19	0.724
Ability	21	0.7216
Activity	25	0.6883
Research	23	0.6595
Data	23	0.6405
Performance	30	0.6272
Effort	15	0.6264
Use	13	0.6241
Healthy control subject	10	0.5985
Patient group	13	0.5659
Association	25	0.5643
Self	11	0.5006
Amotivation	16	0.4898
Treatment	24	0.4866
Apathy	19	0.4686
Relationship	41	0.4345
Healthy control	36	0.4177
Reward	52	0.414
Reinforcement learning	13	0.4091
Positive symptom	11	0.3934
Context	16	0.3731
Schizophrenia patient	25	0.3134
Evidence	21	0.2737
Age	19	0.2159
Background	21	0.131

### Research over time

#### Growth and trends of productivity

**Figure 7:**
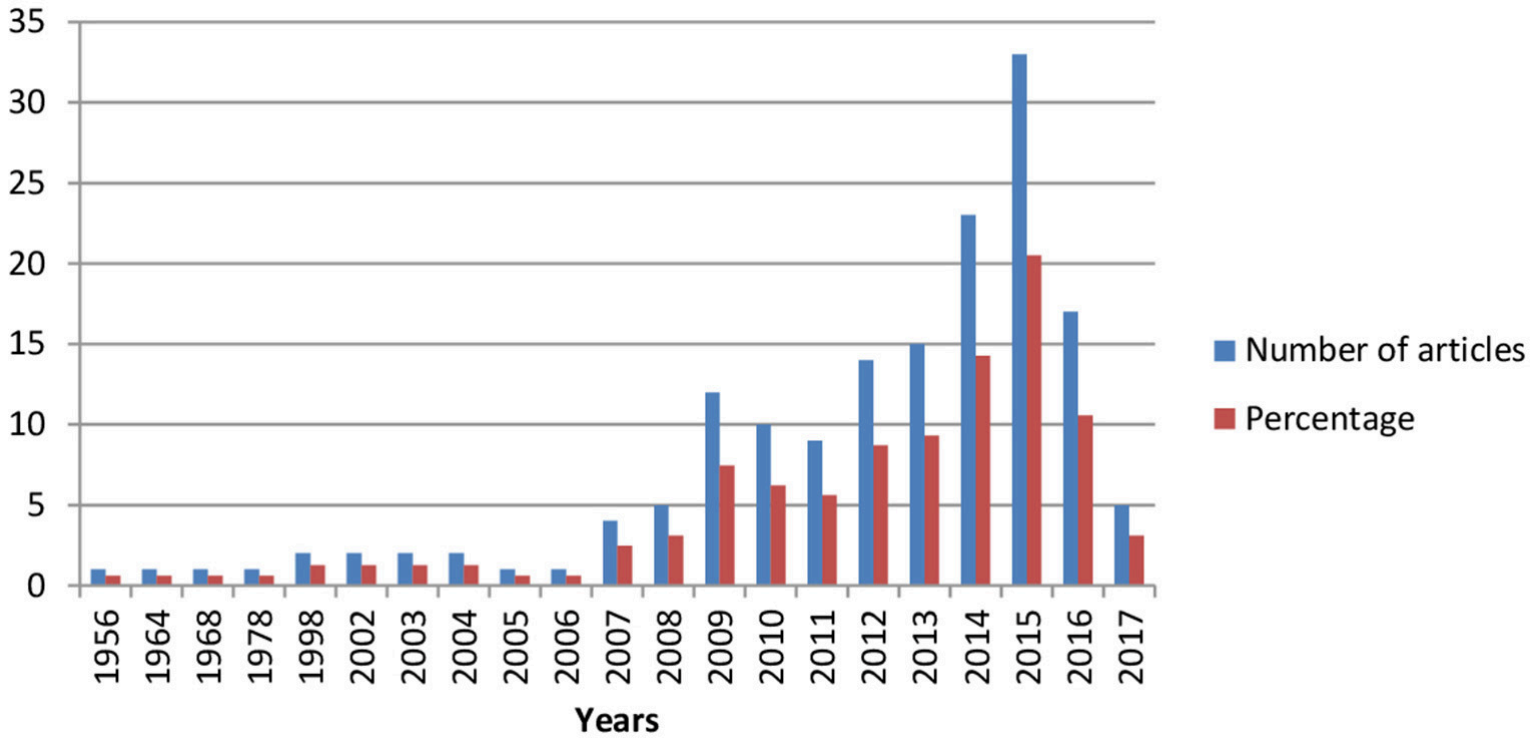
Number of articles published by year.

As can be seen in Figure 7, literature production was scant during the first decades of the period considered: only four articles (2.48% of the total) were published between 1956 and 1978, after which a further eight (4.96%) appeared between 1998 and 2004, before another decline in interest in 2005 and 2006.The number of articles published on motivation in schizophrenia then increased steadily from 2007 to 2015, with production in the latter year accounting for 20.49% of the studies included in this study. However, output then fell in 2016 and again in 2017 (up until November), a year in which the number of articles published (i.e., five) was only 29.4% of the total published the previous year.

**Figure 8:**
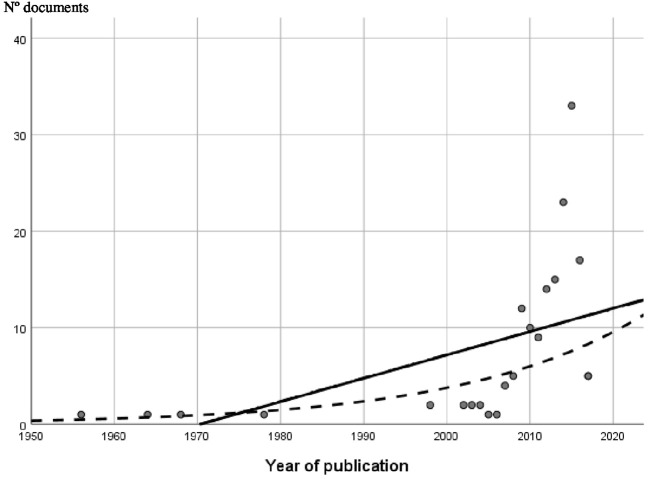
Price's law adjustment.

Linear, exponential, and logistic regression models were fitted in order to test whether the data followed Price's law. All three models were statistically significant but explained a different proportion of the variance: linear (*R*^2^ = 0.262), logistic (*R*^2^ = 0.261), and exponential (*R*^2^ = 0.499). Thus, the logistic and exponential models provided the best fit to the data (see Figure 8).

### Change in terminology over time

As can be seen in Table 6, since the late 1990s there has been an increase in the diversity of terms used to refer to motivation in schizophrenia. IM was first analyzed in 1956, but it was not until 2008 that research into this specific form of motivation resumed. The years in which most studies of IM in schizophrenia were published were 2010, 2014, and 2015. By contrast, SE was first analyzed in 2004 and appeared intermittently in the literature thereafter, reaching a peak in 2013. RR has been the most widely studied term of motivation in schizophrenia since 1964 and has been addressed in a steadily growing number of studies throughout the period considered for this study; the largest number of studies was published in 2015. The first published evidence about AP in schizophrenia to appear in this study dates from 2003, although research interest then remained stable until 2015, the year in which most studies were published. AV has received less attention; the first recorded study to address AV was published in 2010, and peak interest was reached in 2015. The study of other motivational terms appeared in 2008, reaching a peak in 2014.

**Table 6:** Pattern over time in the number of articles using different motivation terminologies.

**Year**	**IM**	**SE**	**AP**	**RR**	**AV**	**O**	**Total**
1956	1						1
1964				1			1
1968				1			1
1978				1			1
1998				2			2
2002				2			2
2003	1		1				2
2004		1	1				2
2005		1					1
2006				1			1
2007		1	1	2			4
2008	2		1	2			5
2009	1	2	1	7		1	12
2010	4	2	1	4	1		12
2011	1		2	6			9
2012	2	2		8	2		14
2013	1	4	5	4	2	2	18
2014	5	1	2	14	1	3	26
2015	5	1	8	16	8	2	40
2016	3	1	3	10	1	1	19
2017	2	1	1	3			9
Total	28	17	27	84	15	9	

*Some works were studying more than one term. IM, Intrinsic Motivation; SE, Self-Efficacy and Defeatist Believes; AP, Apathy; RR, Rewards or Reinforcements; AV, Avolition; O, other terms of motivation*.

### Use of different approaches to the study of motivation over time

Table 7 shows the pattern over time in the use of different methodologies to study motivation in schizophrenia. The diversity of instruments has clearly increased over time, particularly since 2007. Brain techniques were introduced at the beginning of 2000 and the number of studies using these techniques then rose steadily, reaching a peak in 2015. Behavioral tasks were introduced in 1956, but fell out of favor in the 1980s, reappearing around 2006. Again, the years in which most behavioral studies were published were 2015 and 2016. Questionnaires have been the most widely used instrument since they were first introduced in 2002, and questionnaire-based studies increased steadily in number up until 2016. The least used instruments to assess motivation in schizophrenia have been those related with the measurement of brain activity.

**Table 7:** Pattern over time in the research methodologies used to assess motivation.

**Year**	**Brain techniques**	**Behavioral tasks**	**Questionnaires**	**Total**
1956		1		1
1964		1		1
1968		1		1
1978		1		1
1998		2		2
2002		2	1	3
2003		1	1	2
2004	1		2	3
2005			1	1
2006	1	1		2
2007	1	2	2	5
2008	1	3	3	7
2009	2	8	6	16
2010	3	6	7	16
2011	1	6	5	12
2012	5	10	5	20
2013	3	7	12	22
2014	6	8	13	27
2015	13	18	24	55
2016	7	13	12	32
2017	2	5	5	13
Total articles	46	96	99	241

## Discussion

We have revised the main trends in the study on motivation in schizophrenia through exhaustive examination of the literature and bibliometric methods. Results showed a significant growth in the number of studies, especially in recent decades, with 20.49% of the studies included in this study being published in 2015. Since the late 1990s, the increase in the number of published studies on motivation in schizophrenia has followed exponential and logistic curves, reflecting the considerable and growing interest in this field (Price, 1963). However, if studies are analyzed according to the different motivation terms they use, then the upward trend in productivity does not apply equally to them all, with the exception of RR, which attracted particular interest in the scientific community between 2009 and 2016 (see Table 6), even though the term has been present for a period of 17 years of research.

The sheer scale of expansion challenges researchers' capacity to conduct a comprehensive analysis of the available motivation terms. Moreover, the concepts and definitions of motivation are complex, interdependent, and interdisciplinary (see Figure 3), yielding publications that may be grouped into three broad areas of interest, namely, neuroscience, psychology, and psychiatry. As evidenced by the results, research has focused particularly on the term “reward” as a means of understanding motivational impairments in schizophrenia (Barch and Dowd, 2010), providing important insights into the cognitive and neural mechanisms associated with these impairments (Strauss et al., 2014). This interest in neural mechanisms is clearly linked to the development of new brain techniques for studying RR, even though behavioral tasks continue to be the most widely used approach (see Table 7). In fact, the most used task to assess reward processing was the Effort Expenditure for Reward Task (EEfRT, 2009), a computerized effort-based decision-making task in which participants are given an opportunity on each trial to choose between two different task difficulty levels in order to obtain monetary rewards: a high effort option and a low effort option (Treadway et al., 2009, 2015).

Self-reports are the preferred instrument in the literature for assessing other associated terms in the study of motivation, such as IM, AP, SE, and AV. The widespread use of questionnaires may reflect the ease with which they can be attained and applied or may imply that there is greater confidence in psychometric measures than in neurophysiological or behavioral tools for assessing motivation. It should be noted, however, that some inconsistent results have emerged when using self-reports. For example, Barch et al. (2008) found that people with schizophrenia did not differ from participants without schizophrenia in two intrinsic motivation domains, as measured by the Motivational Trait Questionnaire (MTQ; Heggestad and Kanfer, 2000). However, Choi et al. (2012) found that patients with schizophrenia reported less IM than did healthy controls when rating their motivation using the Intrinsic Motivation Inventory (IMI) adapted for people with schizophrenia. These results illustrate how two instruments measuring the same construct are not properly adapted for people with mental disabilities like schizophrenia. In this respect, some authors have recently highlighted that only a small number of motivation self-reports are appropriate for patients with severe mental illness (Cooper et al., 2015). Despite this, there has been an increase in the use of different and new questionnaires and behavioral tasks to measure motivation. In addition, the set of terms used to refer to motivation has increased in recent years, so not only are more instruments required to measure different and specific motivational processes, but further efforts are needed to adapt these measures to people with mental disorders and their specific cognitive disabilities.

The least widely used concept in the study of motivation in schizophrenia is AV, the first study of which was published in 2010, the same year as the review by Foussias and Remington (2010), who in discussing two sub-domains of negative symptoms used the term “avolition” to refer to the motivational deficits observed in schizophrenia. However, this term has been slow to catch on, and AP seems to be preferred, even though AP and AV are often used as synonyms in the literature on schizophrenia (Bortolon et al., 2017).

Our evaluation of worldwide trends in research productivity in the field of motivation in schizophrenia over 61 years of study indicates that North America produces the most reports: the USA and Canada were the countries with the greatest volume of published research over the period studied, jointly accounting for more than 50% of the total output inspected. Among the other continents, Europe made a significant contribution, with the UK and Germany being the largest producers.

Given that institutional, national, and international research collaborations are directly related with the number of citations a publication receives, and that collaborations therefore play a key role in the main research agendas (Gazni and Thelwall, 2014; Hassan and Haddawy, 2015), the general index of collaboration in the study of motivation in schizophrenia (only 15.84% of the studies included) suggests that authors do not establish sufficient contact with their international colleagues. Of the two most productive continents referred to above, Europe generated more collaborative studies than North America. The country with the greatest research output was the USA, which recorded the largest number of collaborations with other countries in absolute terms (20); however, among the four most productive countries, the UK recorded the highest rate of collaboration (70.58% of published studies), followed by Germany (30.00%), Canada (28.57%), and the USA (24.69%). These data suggest that scientific production is centralized by countries and that professionals might not be benefiting from sharing and integrating procedures and knowledge generated by other groups. This pattern of production may be impeding further development of this research area.

Consistent with the literature (Qasim, 2016), we identified a large number of publications with a small number of citations per publication, and a small number of publications with a very high number of citations (32 studies with 16 or more citations). This illustrates the interest shown by the scientific community in the work of a group of authors (primarily from the USA and Germany) who are investigating the mechanisms and processes of reward dysfunction, a topic that accounted for 56.25% of the highly cited publications. The network derived from the co-citation analysis by author indicates that motivation in schizophrenia is a focus of research interest for authors working in three distinct but closely related areas, namely Neurocognitive function cluster, Reward processing cluster, and Psychiatry and Clinical approach cluster. It can be seen in Figure 5 that the co-citation relationship is stronger between researchers working in the first two of these areas; however, all three areas are involved in the enhancement of the functional outcomes in schizophrenia

The network resulting from the co-citation analysis by journal shows a strong tendency for co-citation between the journals *Schizophrenia Research* and *Schizophrenia Bulletin* (see Figure 3). This is consistent with the results for journal productivity, since these two journals accounted for 30.02% of the total literature production; *Schizophrenia Research* was the most productive journal and *Schizophrenia Bulletin* was among the top five. The network derived from the co-citation analysis by cited reference (see Figure 4) shows that the output of these two journals forms two clusters which we label “Cognition” and “Reward/Neuroimaging,” this being consistent with the term most widely used in studies of motivation in schizophrenia, namely RR. Note that the most oft-cited reference is Kay et al. (1987) a publication that provides the basis for current conceptualizations of negative symptoms in schizophrenia and methods for assessing them. Similar to previous findings (Qasim, 2016; Flis and van Eck, 2017), the analysis of the co-occurrence of terms showed the terms that best capture the literature in our revised topic. Our analysis revealed two main clusters of terms (see Figure 6). The dense area in red (Functional assessment cluster) encompasses terms related to performance, assessment, and functional outcomes. The cluster in green (Reward processing cluster) has the term “reward” at its center. This central position, coupled with the size of the corresponding node, indicates that it is this term which best reflects the co-occurrence of terms, and underlines that reward is the main dimension studied by the publications included in this study.

Research production is centralized by the fact that the majority of articles are restricted to a small number of journals and a small group of researchers. Although it is often considered that psychology, psychiatry, and neuroscience studies are well connected, our co-citation analysis identified opportunities for greater inter-disciplinary collaboration with biological and neuroimaging approaches, and between authors. As has been noted elsewhere, this is important, as a failure to consider work from other disciplines could lead to partial or incorrect conclusions (Stuckler et al., 2015).

Due to the heterogeneity in measurement methods, research designs, and ouitcome variables, it was not possible to perform a meta-analysis or to calculate pooled effect sizes. Further, we restricted our analysis to quantitative studies in order to facilitate the codification and generalization of data, although qualitative studies can also provide useful insights. Despite these limitations, the present study highlights the need to use standardized instruments and terminologies when designing studies on motivation in schizophrenia. Moreover, the field will also benefit from an increase in the level of collaboration between the various approaches within the psychological and psychiatry fields, and hence between authors from different countries.

## Author contributions

All authors certify that they have participated sufficiently in the work to take public responsibility for the content, including participation in the concept, design, acquisition of data and writing of manuscript draft (AN-G, JG-B), codification of data, analysis and writing of the final manuscript (VC, AN-G), and revision of the manuscript (JG-B, VC).

### Conflict of interest statement

The authors declare that the research was conducted in the absence of any commercial or financial relationships that could be construed as a potential conflict of interest.
